# Influence of Global Atmospheric Change on the Feeding Behavior and Growth Performance of a Mammalian Herbivore, *Microtus ochrogaster*

**DOI:** 10.1371/journal.pone.0072717

**Published:** 2013-08-19

**Authors:** Christopher W. Habeck, Richard L. Lindroth

**Affiliations:** 1 Department of Zoology, University of Wisconsin, Madison, Wisconsin, United States of America; 2 Department of Biology, Kutztown University, Kutztown, Pennsylvania, United States of America; 3 Department of Entomology, University of Wisconsin, Madison, Wisconsin, United States of America; INRA-UPMC, France

## Abstract

Global atmospheric change is influencing the quality of plants as a resource for herbivores. We investigated the impacts of elevated carbon dioxide (CO_2_) and ozone (O_3_) on the phytochemistry of two forbs, *Solidago canadensis* and *Taraxacum officinale*, and the subsequent feeding behavior and growth performance of weanling prairie voles (*Microtus ochrogaster*) feeding on those plants. Plants for the chemical analyses and feeding trials were harvested from the understory of control (ambient air), elevated CO_2_ (560 µl CO_2_ l^−1^), and elevated O_3_ (ambient × 1.5) rings at the Aspen FACE (Free Air CO_2_ Enrichment) site near Rhinelander, Wisconsin. We assigned individual voles to receive plants from only one FACE ring and recorded plant consumption and weanling body mass for seven days. Elevated CO_2_ and O_3_ altered the foliar chemistry of both forbs, but only female weanling voles on the O_3_ diet showed negative responses to these changes. Elevated CO_2_ increased the fiber fractions of both plant species, whereas O_3_ fumigation elicited strong responses among many phytochemical components, most notably increasing the carbon-to-nitrogen ratio by 40% and decreasing N by 26%. Consumption did not differ between plant species or among fumigation treatments. Male voles were unaffected by the fumigation treatments, whereas female voles grew 36% less than controls when fed O_3_-grown plants. These results demonstrate that global atmospheric change has the potential to affect the performance of a mammalian herbivore through changes in plant chemistry.

## Introduction

Concentrations of atmospheric CO_2_ and tropospheric O_3_ have increased rapidly since the beginning of the industrial revolution and this trend is expected to continue if mitigating actions are not implemented [1]. Global atmospheric changes (GAC), in particular, increased atmospheric CO_2_ and tropospheric O_3_, are eliciting physiological changes in plants that affect, often negatively, the nutritive quality of plant tissues [2]–[4]. Plants grown under elevated CO_2_ often have tougher leaves, higher concentrations of carbon-based compounds (e.g., carbohydrates, fiber, and tannins) and lower concentrations of nutrients, particularly nitrogen [2]. Although many studies and several meta-analyses are published on the effects of tropospheric O_3_ on tree foliar quality [3],[5],[6], there are fewer accounts for herbaceous species (but see [7]). Ozone enters the stomata and damages components of the photosynthetic pathway (e.g., chlorophyll and rubisco) thus reducing the rate of carbohydrate production and accumulation in trees [3],[4]. Concentrations of phenolic compounds often, but not invariably, increase in response to O_3_ [3],[4],[7]. For both grasses and forbs exposed to O_3_, the available literature suggests a trend towards increased cellulose and lignin, but variable responses of nitrogen concentration [8]–[17]. A general reduction in plant quality due to GAC is expected to have significant impacts on herbivore populations by reducing growth rate, fecundity, or survival.

Generalist herbivores can modify their behavior and/or physiology to compensate for short-term changes in plant quality, for example through diet switching or changes in gut morphology [18]–[20]. Yet we know relatively little about how herbivores will respond to a consistent decline in plant quality mediated by predicted changes in atmospheric gas concentration and climate. Many short-term studies with phytophagous insects show that both CO_2_- and O_3_-mediated changes in plant chemistry impact insect growth and development, regardless of any compensatory actions taken by the consumer [2],[4],[21]. To our knowledge, however, there are no published accounts on the growth response of herbivorous mammals to these GAC-mediated changes in plant quality. Nonetheless, several studies have assessed the indirect effects of atmospheric change on the symbiotic gut microfauna of ruminant mammals, using an indirect *in vitro* method [14],[22]–[24]. The data suggest that *in vitro* dry matter digestibility (IVDMD) decreases for grasses and forbs when CO_2_ concentration is double ambient levels [10],[17],[23]–[25], but no difference occurs at lower levels (e.g., ambient × 1.5) [14],[22]. If microbial digestion in ruminants is indicative of a general response, then it is reasonable to assume that other mammals with a significant reliance on microbial fermentation (e.g., voles, lemmings, and rabbits) will be negatively impacted by global atmospheric change.

Voles and other small herbivorous mammals can respond to reductions in plant quality by increasing total consumption [26], modifying relative consumption among plant species [27], increasing gut volume or surface area [19],[26],[28], or employing a combination of these strategies [18]. Negative impacts on herbivore growth will be realized, however, if these actions are not sufficient to compensate for reductions in plant quality.

Prairie voles are generalist herbivores whose diet consists mainly of the aboveground portions of herbaceous plants during the growing season [29]. Food selectivity is dependent on the quality of the plant, which is positively correlated with digestibility and nutrient concentration (e.g., nitrogen), and negatively correlated with dietary fiber and plant defenses [29]–[31]. The ceacum in many voles, including prairie voles, is large compared to most other muroid rodents [32]. The functional significance of an enlarged ceacum in *Microtus* spp. is unconfirmed, but is likely a morphological adaptation to increase digestibility of their herbaceous diet [29],[32]. In fact, regardless of their small size, voles and ruminants have similar coefficients of dry matter digestibility for several forage species [33]–[35]. Because they are relatively responsive to the chemical changes typical of plants grown in elevated CO_2_ and O_3_, voles are an appealing model organism for exploring the response of mammalian herbivores to global atmospheric change.

In this study, we used forbs harvested from the Aspen FACE facility near Rhinelander, Wisconsin USA and prairie voles to test three hypotheses about the effects of GAC on plant nutritive quality and mammalian herbivore behavior and performance: (1) elevated CO_2_ and O_3_ will modify the chemical composition of plants such that quality as a food resource will be reduced, (2) prairie voles will compensate for reductions in plant quality by increasing total consumption and by modifying the proportional consumption of *Solidago* and *Taraxacum* to maximize the quantity and quality of nutrients in their diet, and (3) the growth rate of weanling voles will be negatively impacted by GAC-mediated changes in plant chemistry.

## Materials and Methods

### Ethics statement

The use of *Microtus ochrogaster* in this study was approved by the Institutional Animal Care and Use Committee (IACUC), Research Animal Resources Center, University of Wisconsin-Madison (Protocol number G00503-1-03-06).

### Experimental Design

With prior approval from the Aspen FACE Steering Committee, we collected *Solidago canadensis* and *Taraxacum officinale* during early July 2007 from the U.S. Department of Energy Aspen FACE (Free Air CO_2_ Enrichment) research facility near Rhinelander, Wisconsin. These species were selected because they are common members of plant communities within the geographic range of prairie voles, are known to be consumed by voles [31] and were reasonably abundant in the experimental plots such that we could harvest an adequate amount of biomass for the feeding trials. Aspen FACE was established in 1997 to evaluate the effects of elevated CO_2_ and O_3_ on the structure and function of northern forest ecosystems. The experimental design for the fumigation treatments was a 2×2 factorial with three blocks. The experiment consisted of four 30-m diameter rings per block, each block containing one ring for each treatment: ambient, elevated CO_2_ (560 µl CO_2_ l^−1^), elevated O_3_ (ambient × 1.5), and elevated CO_2_ + O_3_. Each ring was divided into three communities: mixed aspen (*Populus tremuloides*) genotypes, aspen-paper birch (*Betula papyrifera*), and aspen-sugar maple (*Acer saccharum*). Plants were collected from the aspen-maple understory of all control, elevated CO_2_, and elevated O_3_ rings, but not from elevated CO_2_ + O_3_ rings. We excluded the CO_2_ + O_3_ treatment because we were limited in our ability to produce enough vole progeny to include all four Aspen FACE treatments. Plants were kept chilled until used in growth trials (i.e., approximately 3hrs after harvest). A subset of these plants was oven-dried for 48 h at 60°C, then ground in a Wiley mill and reserved for plant chemical analysis. Samples were stored at –20°C until analyzed.

### Plant Chemistry

We assayed several chemical parameters of plants to quantify the effect of GAC on plant quality. These were carbon (C), nitrogen (N), total non-structural carbohydrates (TNC; the sum of soluble sugars and starch), fiber, lignin, and protein-binding capacity (an index of plant defense). Carbon and nitrogen were quantified using a Thermo Finnigan Flash 1112 elemental analyzer (Thermo Finnigan, San Jose, CA, USA). Sugars and starch were quantified spectrophotometrically using a dinitrosalicylic assay, as modified by [36]. Fiber (cellulose and lignin) and lignin were quantified as acid detergent fiber (ADF) and acid detergent lignin (ADL), respectively, via sequential extraction in hot acid-detergent using an Ankom 200 Digestor (ANKOM Technology Corporation, Fairport, NY). Protein binding-compounds were assayed using the polyethelene glycol (PEG) incubation technique described by [37]. The PEG assay is an index of plant defense and quantifies the effect of all protein-binding compounds (mostly tannins and other phenolic compounds) found in a plant sample on *in vitro* dry matter (IVDMD) and *in vitro* nitrogen (IVND) digestibility for hindgut-fermenting herbivores. The difference in percent digestibility between PEG-incubated and control-incubated samples indicates the reducing power (RP) of protein-binding compounds on dry matter (RP-DM) and nitrogen (RP-N) digestibility.

### Feeding Trials

We used weanling progeny of wild-caught prairie voles for the feeding assays. We focus on weanlings because growth post-weaning is a fundamental metric of vole performance and a generally recognized proxy for vole fitness. For example, growth rates can influence population dynamics by dictating the timing and success of dispersal and reproduction, and the likelihood of survival through winter [38]–[40]. During May 2007, adult prairie voles were live-trapped from an alfalfa field in northwestern Illinois (42°11’N, 90°13’W) and transported to animal rooms at the University of Wisconsin. Voles were maintained as single breeding pairs in shoebox cages at 23°C on a 14L:10D photoperiod. Each breeding pair was maintained until the female gave birth, after which the breeding male was removed. The progeny of wild-caught voles were removed from their mothers 21 days post-partum. Weanlings were kept on the normal laboratory diet (Harlan Teklad Diet 7778) for at least three days prior to the experiments and their mass was monitored to ensure that they were performing well on solid diet. No voles lost >10% body mass during the pre-trial period.

At the start of the growth experiment, weanling voles were weighed and individually housed in shoebox cages with *ad lib* water, a maintenance level of laboratory diet assuming a basal metabolic rate (kJ day^−1^) of 3.69*M ^0.601^ (where *M* is body mass [g]) [41], and the treatment diet. The amount of lab diet provided to each vole was calculated using the metabolizable energy content reported by the manufacturer (10.3 kJ g^−1^). Although the value reported is based on the metabolizable energy extracted by woodchucks, this value should be a close approximation of the energy available to voles feeding on this diet. We provided this maintenance level of lab diet because voles generally perform poorly on plant diets of limited diversity, as was the case in this study. Also, providing voles a maintenance level of lab diet ensured that any growth observed was due primarily to the consumption of the plant-based treatment diets. The treatment diet consisted of leaves from both *Solidago* and *Taraxacum* in excess. Fresh treatment diet was weighed and distributed so that plants from each FACE ring were given exclusively to each of three voles (9 rings×3 voles  =  27 total voles). We used voles from eight litters in the experiment. Litter sizes ranged from two to seven voles. To reduce the potential for genetic and maternal effects to skew results, voles from the same litter were distributed across fumigation treatments, and in no case were two voles of the same litter assigned to receive treatment diet from the same replicate FACE ring. After 24 h, the orts were removed and replaced with fresh plants. After a three-day acclimation period, daily plant consumption and body mass were recorded for seven days (hereafter, one week). Although a longer trial period would have been preferable, the duration of our trials was limited by the availability of plant biomass in the understory of the FACE rings. Daily plant consumption (dry matter intake) for each species was quantified gravimetrically as the difference between the amount provided and the orts remaining after 24 h. We used a subset of the plant material harvested for the feeding trails to quantify the dry mass provided during the trials. Dry mass provided was calculated as the product of fresh mass given and % dry mass, where the % dry mass for each species was quantified for each individual FACE ring. We quantified proportional consumption as the consumption of each species, relative to total consumption. The growth rate response of weanling voles was calculated as change in body mass after one week on the treatment diets. Chemical analysis of the orts was not conducted due to contamination by feces and urine. However, we believe that the chemical composition of the orts is probably indistinguishable from that of the total plant material provided to the voles as we observed no selection for particular tissues. Therefore, the chemical characteristics of the plant material provided represent a reasonable approximation of the constituents consumed, and inferences are made within that context.

### Statistical Analysis

Split-plot analysis of variance (ANOVA) was used to test the effects of: 1) fumigation treatment and plant species on foliar chemistry (C:N, N, TNC, ADF, ADL, ADF:N, ADL:N, IVDMD, IVND, RP-DM, RP-N), and 2) fumigation and vole sex on plant total consumption, plant proportional consumption, and vole growth rate. Because the CO_2_ + O_3_ treatment was not included in our experiment, we could not assess the effects of CO_2_ and O_3_ as separate fixed factors within a single model. Instead, we evaluated the effects of fumigation as a single factor with three levels (control, elevated CO_2_, and elevated O_3_). Fumigation treatment was the whole-plot factor for all the response variables. Degrees of freedom for F tests were assigned using the Satterthwaite approximation. Replication at the whole-plot level at Aspen FACE is low (n = 3), increasing the probability of type II errors. Therefore, we report *P*-values <0.10 as significant [42]. Because we were interested in within- and among-species responses to the treatments, and how levels within treatments contributed to this response, we used Tukey’s HSD test for *post-hoc* pairwise comparisons of group means when there were significant main effect and interaction terms from the full model [43]. As with the main model, we interpret *P*-values <0.10 from *post-hoc* analysis as statistically significant.

We used partial least squares regression (PLSR) analysis to investigate relationships of phytochemicals to total plant consumption and growth rate. The benefit of using PLSR over other statistical procedures commonly used to relate response variables to multiple predictor variables (e.g., multiple linear regression) is that PLSR is robust to deficiencies commonly associated with ecological data, specifically low observation to predictor variable ratios and multicolliniarity among predictor variables [44], as is typical of plant chemistry data. PLSR is a data reduction technique that reduces many (potentially correlated) predictor variables to fewer orthogonal latent variables that maximize the explained variance in the response variable [45]. The number of latent variables produced by PLSR for a particular data set can equal the number of original predictor variables. However, including all latent variables in a model can lead to over-fitting [46]. We used cross validation to indicate the number of latent variables to retain. Cross validation is an iterative process that selects the optimum number of latent variables to include in the model via their additive effect on the predictive residual sum of squares (PRESS). If adding an additional latent variable to the model does not reduce PRESS, the preceding number of latent variables is retained [46]. In addition, we reduced the number of phytochemical variables in the model based on their contribution to explained variance in the feeding and growth rate response of voles using the variable importance for the projection (VIP) method. Phytochemical variables with a VIP < 1.0 were excluded as they indicate a marginal influence on the final model [47]. Finally, a linear regression model was built using the retained extracted factors. The size and sign of the regression coefficients indicate the magnitude and direction of the influence of the individual phytochemicals on the vole response. Predictor and response variables were scaled and centered to unit variance, ensuring that all variables were weighted relative to their contribution to variation in the data and that selection of factors was based on the amount of variation they explain. We examined the relationship between the response variable and the predicted response derived from the final PLSR model using general linear regression. Any model with *P*-values ≥0.05 was excluded from further inference. Because voles had simultaneous access to both plant species during the feeding trials, we created weighted averages for the phytochemical variables before relating them to the feeding and growth rate response of each vole (n =  27 voles). Weighted averages were computed as the sum of the products of percent consumption for each vole and percent concentration of phytochemicals for each of the two plant species. All statistical analyses were conducted using JMP Version 9 statistical software (SAS Institute Inc., Cary, NC).

## Results

### Effects of CO_2_ and O_3_ on plant chemistry

**Figure 1:**
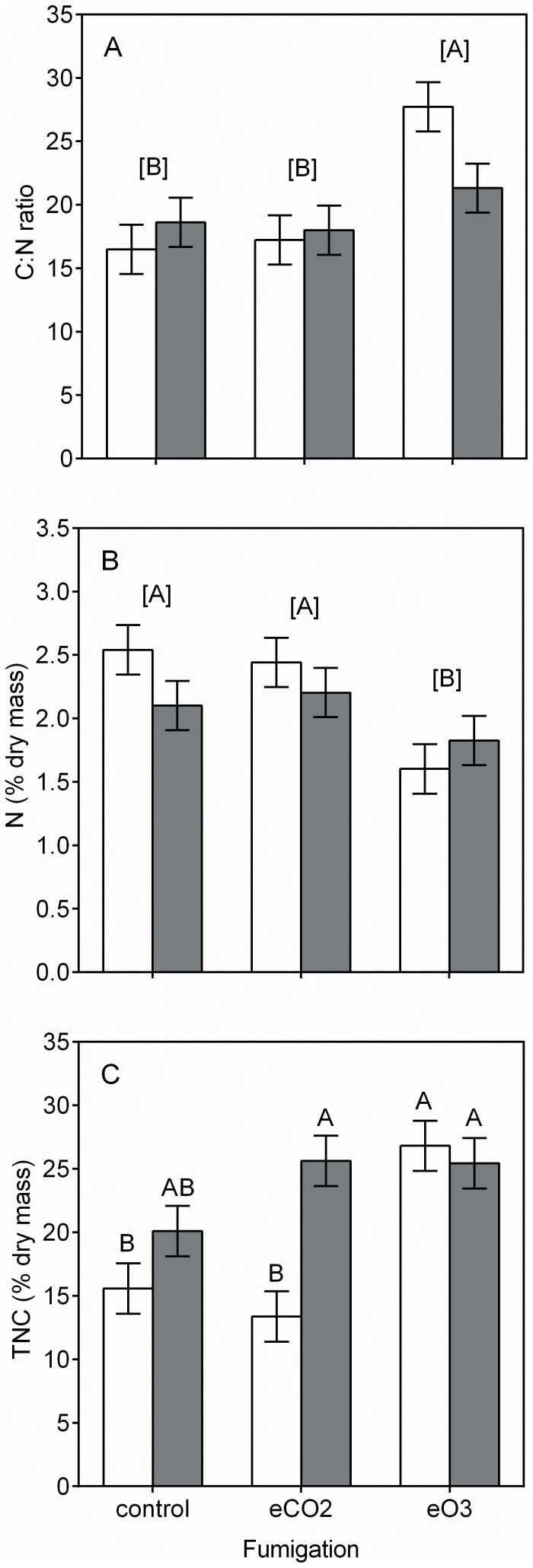
Fumigation and plant species effects on a) C:N, b) N, and c) TNC. White and gray bars indicate least square means ±SE (n = 3 replicate FACE rings for each group mean shown) for *S. candensis* and *T. officinale*, respectively. Significant differences between group means were analyzed using Tukey’s HSD *post hoc* test, where differences between group means are indicated by letters within brackets (e.g., “[A]”) for fumigation main effects, and pairwise comparisons among levels of fumigation and plant species are indicated by letters without brackets (e.g., “A”) when there were significant interaction terms from the main model.

**Figure 2:**
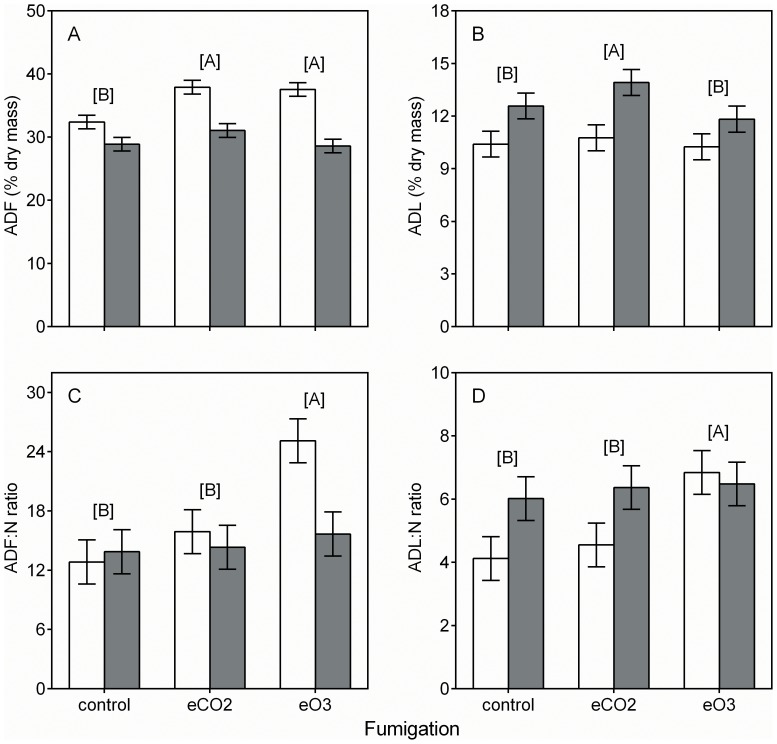
Fumigation and plant species effects on a) ADF, b) ADL, c) ADF:N, and d) ADL:N. White and gray bars indicate least square means ±SE (n = 3 replicate FACE rings for each group mean shown) for *S. candensis* and *T. officinale*, respectively. Figure format is as described for Figure 1.

**Figure 3:**
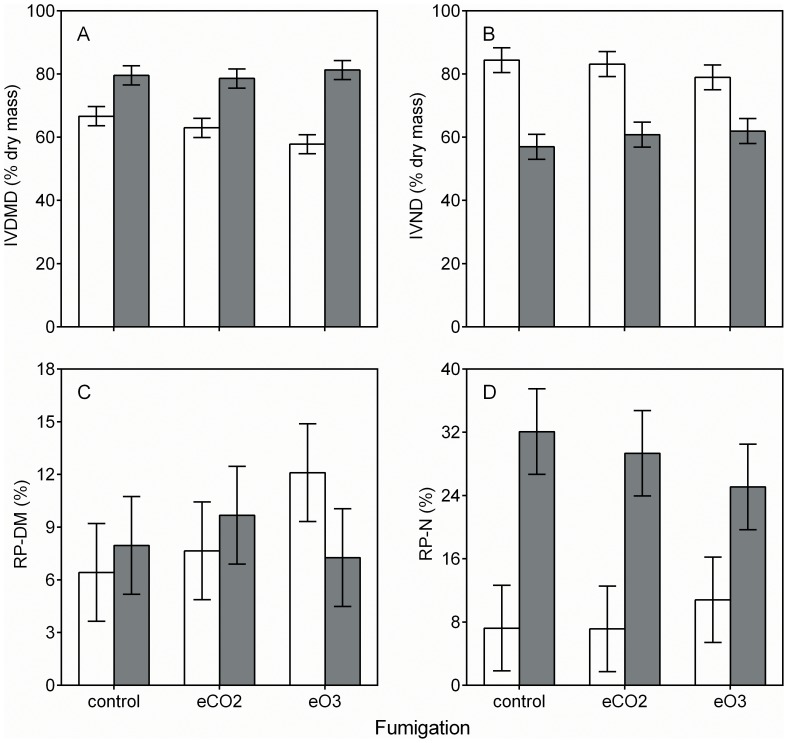
Fumigation and plant species effects on IVDMD, IVND, RP-DM, and RP-N. White and gray bars indicate least square means ±SE (n = 3 replicate FACE rings for each group mean shown) for *S. candensis* and *T. officinale*, respectively. Means for IVDMD, IVND, and RP-N, but not RP-DM differ between species (*P*  =  <0.05). Split-plot ANOVA results for these data are shown in Table 1.

The phytochemistry and digestibility of *Solidago* and *Taraxacum* were influenced by the fumigation treatments (Table 1). Based on *post hoc* analysis, no plant species × CO_2_ fumigation treatment interactions occurred for the plant constituents assayed, suggesting that these two species respond in a similar fashion to elevated CO_2_. Elevated CO_2_ had no effect on levels of C:N, N, TNC, IVDMD, IVND, RP-DM, or RP-N (Figure 1, Figure 2). Elevated CO_2_ increased ADF and ADL by 13% and 7%, respectively (Figure 2 A and B). Ozone fumigation did not affect levels of ADL, IVDMD, IVND, RP-DM, or RP-N (Figure 2 A, Figure 3). Elevated O_3_ increased plant C:N 40% (Figure 1 A). This response was driven by a 26% decrease in N and a 46% increase in nonstructural carbohydrates (i.e, the sum of starch and soluble sugars; Figure 1 B and C). Elevated O_3_ increased ADF, ADF:N, and ADL:N by 8%, 52%, and 31%, respectively (Figure 2 A, C and D).

**Table 1:** Fumigation, species, and fumigation × species effects for chemical parameters of *S. canandensis* and *Taraxacum* grown under control, elevated CO_2_, or elevated O_3_.

	Fumigation			Species			F × S		
Response variable^1^	df	*F*	*P*	df	*F*	*P*	df	*F*	*P*
C:N	2,6	7.52	0.023	1,6	0.63	0.457	2,6	3.29	0.109
N	2,6	4.75	0.058	1,6	1.44	0.275	2,6	2.43	0.169
TNC	2,6	20.88	0.002	1,6	6.59	0.043	2,6	3.89	0.082
ADF	2,6	19.81	0.002	1,6	31.91	0.001	2,6	1.94	0.224
ADL	2,6	11.55	0.009	1,6	7.78	0.032	2,6	0.31	0.744
ADF:N	2,6	5.81	0.039	1,6	3.13	0.127	2,6	2.79	0.139
ADL:N	2,6	4.75	0.058	1,6	2.8	0.145	2,6	1.23	0.356
IVDMD	2,6	1.11	0.390	1,6	36.87	0.001	2,6	1.22	0.358
IVND	2,6	0.06	0.945	1,6	112.65	<0.001	2,6	2.06	0.209
RP-DM	2,6	0.44	0.665	1,6	0.03	0.863	2,6	0.88	0.461
RP-N	2,6	0.04	0.962	1,6	38.33	0.001	2,6	0.92	0.447

1 C:N  =  carbon-nitrogen ratio, N  =  nitrogen concentration, TNC  =  total nonstructural carbohydrate concentration, ADF  =  acid detergent fiber concentration, ADL  =  acid detergent lignin concentration, IVDMD  =  *in vitro* dry matter digestibility, IVND  =  *in vitro* nitrogen digestibility, RP-DM  =  difference in percent digestibility of IVDMD by protein-binding compounds within the plant, RP-N  =  difference in percent digestibility of IVND by protein-binding compounds within the plant.

Although fumigation had no effect on digestibility, both IVDMD and IVND differed between species. Relative to *Taraxacum*, IVDMD was 22% lower and IVND was 37% higher in *Solidago* (Figure 3 A and B). Similarly, fumigation had no effect on RP-DM or RP-N (Figure 3 C and D). However, RP-N differed by species, being 244% higher in *Solidago* relative to *Taraxacum* (Figure 3 D).

### Effects of CO_2_ and O_3_ on consumption

The laboratory chow, provided to ensure that each vole had sufficient food to cover basal energy needs, was completely consumed in every feeding trial. Neither CO_2_ nor O_3_ fumigation, however, influenced the total amount of treatment diet consumed or the proportional consumption of species (Table 2, Table 3). *Solidago* and *Taraxacum* were consumed in equal amounts, regardless of fumigation treatment or vole sex.

**Table 2:** Fumigation, sex, and fumigation × sex effects for vole consumption and growth variables in relation to plant-based diets grown under control, elevated CO_2_, or elevated O_3_.

	Fumigation			Sex			F *×* S		
Response variable	df	*F*	*P*	df	*F*	*P*	df	*F*	*P*
Consumption rate	2,4.1	1.02	0.436	1,13	2.04	0.177	2,11.5	0.29	0.755
Proportional consumption	2,5.4	0.52	0.619	1,20.1	0.78	0.388	2,19.4	0.17	0.847
Growth rate	2,3.4	6.23	0.073	1,21	53.3	<0.001	2,20.3	4.88	0.019

**Table 3:** Mean consumption rate and proportional consumption of *Solidago* and *Taraxacum* (± 1 standard error) by weanling prairie voles for plants grown under control, elevated CO_2_, or elevated O_3_.

	Fumigation treatment		
Response variable^1^	Control	CO_2_	O_3_
Consumption rate (g DM day^−1^)			
Males	6.54 (±0.61)	5.79 (±0.53)	5.38 (±0.61)
Females	5.16 (±0.53)	4.83 (±0.61)	5.16 (±0.73)
Proportional consumption			
Males	0.53 (±0.08)	0.59 (±0.07)	0.49 (±0.07)
Females	0.51 (±0.07)	0.48 (±0.08)	0.45 (±0.08)

1 Because proportional consumption between two species sum to 1.0, we show proportional consumption results for only *Solidago*.

### Effects of CO_2_ and O_3_ on vole growth rate

**Figure 4:**
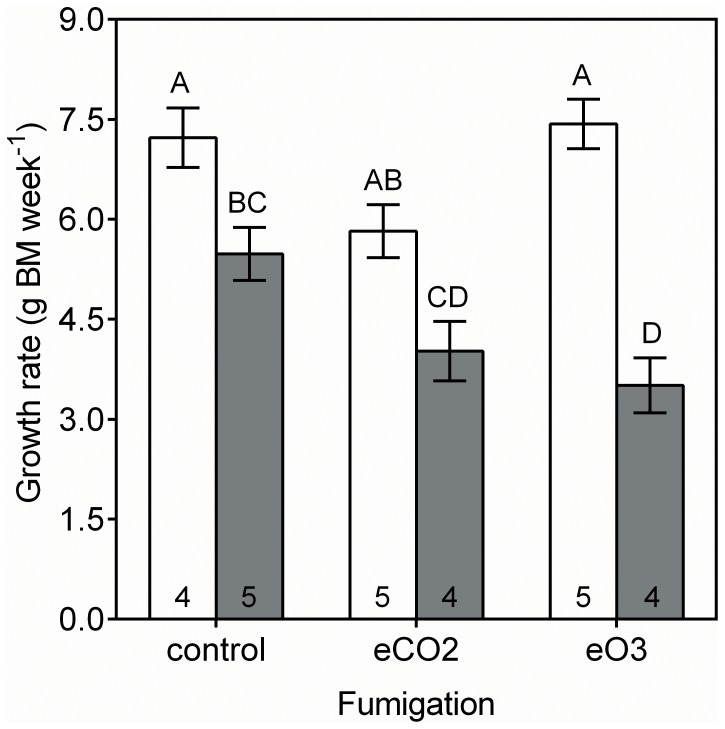
Fumigation and sex effects on weanling vole growth rate. White and gray bars indicate least square means ±SE for male and female voles, respectively. Sample sizes (i.e., replicate voles) for each group mean are indicated inside bars. Significant differences between group means were analyzed using Tukey’s HSD *post hoc* test, where differences between group means are indicated by different letters.

Body mass of weanling prairie voles differed among fumigation treatments after one week; however, the response differed between sexes. Male voles showed no significant response to the fumigation treatments. Females, however, grew 36% less when fed plants harvested from the understory of O_3_ rings (Table 2, Figure 4).

### Relationship of phytochemicals to vole consumption and growth rate

Based on PLSR analysis, several phytochemicals were related to plant consumption and growth rate (Table 4, Figure S1). Total plant consumption by male voles was not related to any of the phytochemicals that we measured. The growth rate of males, however, was negatively associated with levels of ADF and ADL, and to a lesser extent, N. Consumption by female voles was negatively associated with ADL and IVDMD, and positively associated with IVND and RP-N. The growth rate of females was positively associated with N, and negatively associated with CN, TNC, ADF, ADF:N, and ADL:N, although all of these associations were small (absolute standardized coefficients <0.15).

**Table 4:** Regression coefficients from partial least squares regression (PLSR) analysis relating plant traits to consumption by female voles and growth by female and male voles.

	Consumption rate	Growth rate	
Plant trait	Female	Male	Female
C:N	na	na	–0.12
N	na	–0.13	0.10
TNC	na	na	–0.11
ADF	na	–0.68	–0.10
ADL	–0.21	–0.26	na
ADF:N	na	na	–0.14
ADL:N	na	na	–0.11
IVDMD	–0.31	na	na
IVND	0.29	na	na
RP-DM	na	na	na
RP-N	0.21	na	na

The model generated by PLRS for observed versus predicted consumption for male voles was non-significant and is not shown.

## Discussion

To our knowledge, this experiment represents the first published account of the effects of global atmospheric change on the growth performance of a mammalian herbivore, mediated through changes in herbaceous plant chemistry. Both elevated CO_2_ and O_3_ altered the foliar chemistry of two forb species, compared with controls. Elevated CO_2_ increased the fiber fractions of both plant species, but otherwise had no effect on the plant constituents we investigated. Ozone fumigation, on the other hand, elicited strong responses among many phytochemical components, and consequently negatively impacted the growth rate of female voles. Despite the strong effects on plant chemistry, O_3_ fumigation did not affect the growth rate of male voles.

### Effects of CO_2_ and O_3_ on plant chemistry

In general, our results support the hypothesis that GAC will impact the phytochemistry of herbaceous plants. Elevated CO_2_, however, had less of an impact on plant chemistry than did the O_3_ treatment. The modest phytochemical response of *Solidago* and *Taraxacum* to the CO_2_ treatment at Aspen-FACE is similar to the response of other non-woody species in studies where the fumigation treatment is less than double ambient levels [14],[48],[49]. For instance, Muntifering et al. [14] studied the phytochemical response of a forb, *Trifolium repens*, also collected from the aspen-maple understory at Aspen-FACE, and found that CO_2_ fumigation had no effect on levels of N, fiber, phenolics, or IVDMD, whereas elevated O_3_ increased lignin and decreased IVDMD. Herbaceous plants grown under double current levels of CO_2_, however, often show significant chemical responses to the fumigation treatments, albeit with considerable variation among species [24],[50],[51].

One possible explanation for why many herbaceous species exhibit minimal or no response to modest increases in CO_2_ (i.e., less than 2 X ambient) may be their proximity to the soil surface. Bazzaz and Williams [52] measured CO_2_ concentrations across a height gradient in a deciduous hardwood forest stand and found that concentrations of CO_2_ were considerably higher near the soil surface (≤1 meter) than within or above the tree canopy. The CO_2_ fumigation treatment at Aspen FACE was 1.5 X ambient (560 µl/l) and specifically targets the forest canopy, rather than being uniformly distributed. It is possible, therefore, that the CO_2_ treatment in our study was insufficient to elicit a response by understory plants whose CO_2_ environment was inherently elevated compared with that of the canopy.

The phytochemical response of *Solidago* and *Taraxacum* to the O_3_ fumigation at Aspen-FACE is consistent with the results of other studies. In this study, nitrogen concentration increased with exposure to O_3_. Bosinger et al. [8], Frei et al. [17], Lewis et al. [13], and Powell et al. [12] also reported O_3_-induced increases in N concentration, whereas others have found decreases [11],[16] or no response [9]. The mechanisms by which O_3_ influences plant N are not well known. The observed reduction of plant N concentration could be an artifact of increased carbohydrate levels (i.e., a dilution effect; Powell et al. [12]) or O_3_-induced reductions in N-containing constituents such as chlorophyll and Rubisco [53], or both. ADF and ADL levels consistently increase in response to O_3_ exposure [9],[10],[12]-[17]. Similar to Lewis et al. [13] and Szantoi et al. [15], however, we observed increases in ADF, but not ADL.

### Effects of CO_2_ and O_3_ on consumption

Insects typically respond to CO_2_-mediated changes in plant quality by increasing consumption [2]. Counter to our expectations, voles did not compensate for CO_2_- or O_3_-mediated reductions in plant quality by modifying total or proportional consumption of plant species. Voles are responsive to changes in fiber concentration, often increasing dry matter intake as fiber increases in the diet [19],[54],[55]. We found modest, but significant changes in ADF and ADL concentrations under elevated CO_2_. However, these changes were probably insufficient to elicit a compensatory feeding response by the voles. The lack of, or modest associations between, vole consumption and fiber fractions support this notion.

We observed large changes in plant chemistry due to the O_3_ treatment, but voles did not modify their intake to compensate for these changes. Plant C:N increased, driven by a decrease in N and an increase in TNC levels. Although O_3_ fumigation did not affect plant fiber concentration, a reduction in N concentration resulted in increased ADF:N and ADL:N ratios. Prairie voles typically increase food intake and the size of their gastro-intestinal tract to compensate for increased energy demands or fiber concentration of their diet [18]. Less is known, however, about whether changes in nutrient concentration alone, with no appreciable change in fiber concentration, can elicit similar behavioral or physiological responses. Trier [56] and Ditchkoff et al. [54] found no differences in dry matter intake by prairie voles when N concentration in the diet was manipulated, whereas several studies found that dry matter intake by voles increased as energy demands [35],[54],[57] or dietary fiber [19],[35],[54],[58] increased. Although consumption by female voles was inversely associated with IVDMD and positively associated with IVND, voles in this study did not compensate for GAC-mediated reductions in plant N concentration by increasing intake. This and other studies [56], [57],[59] support the idea posited by Karasov and Martinez del Rio [60] that animals preferentially regulate energy intake over other dietary components (e.g., nutrients), regardless of costs to growth. As such, the higher carbohydrate levels in the O_3_-fumigated plants may have limited the ability of voles to compensate for lower plant N concentration.

### Effects of CO_2_ and O_3_ on vole growth rate

The data from this study support the hypothesis that GAC - in this instance, altered tropospheric O_3_ concentration - has the potential to negatively impact the growth rate of mammalian herbivores though changes in plant quality. Female prairie voles gained significantly less mass when fed O_3_-fumigated plants compared to controls. This response was coincident with a negative association between female growth rate and carbon-rich compounds (i.e., ADF, TNC). Contrary to the response of females, the growth rate of weanling males did not differ among fumigation treatments, yet there was a strong negative relationship between male growth rate and ADF and ADL. Most research on the nutritional and developmental ecology of voles does not report (or is not designed to test) differences between sexes. Lindroth et al. [61] found similar growth patterns between adult male and female prairie voles on two synthetic diets, suggesting that accounting for sex in growth studies is unimportant when working with this species. Nonetheless, in our study, initial masses were similar between sexes, yet the overall growth rate of females averaged across all fumigation treatments was 36% less than males. In adult prairie voles, there are physiological and compositional differences between sexes that are likely related to reproductive investment [62]. Whether these sex-related differences are manifest at the weanling stage is currently unknown. However, in light of the results from this study, the potential idiosyncratic growth responses between male and female prairie voles to plant quality deserve attention.

The observed changes in plant chemistry in response to tropospheric O_3_ treatment were substantial (>25% change in concentrations of many phytochemicals), but a clear understanding of how much change in any one chemical component is necessary to elicit a response in weanling prairie voles is currently lacking. However, feeding trials with adult prairie voles suggest that these animals are highly tolerant to extreme changes in plant quality. Ditchkoff et al. [57] showed that adult prairie voles can maintain body mass on diets with a N concentration as little as 1%, whereas Castle and Wunder [35] showed that diets with >80% fiber did not affect the body mass of adult prairie voles. The ability of weanling voles to tolerate extreme changes in plant quality is currently unknown, but should be considerably lower given that the weanling stage is a period of rapid growth in the life history of voles. Further, understanding dietary thresholds necessary to elicit a response in voles (or any animal) is complicated by the coincident change of many phytochemicals to single or multiple environmental drivers.

Previous work on the response of mammals to GAC-mediated changes in plant chemistry have focused on preference trials [63],[64] or indirect measures of performance [14],[22]–[24],[50]. Mattson et al. [64] found that Eurasian hares (*Lepus timidus*) and eastern cottontail rabbits (*Sylvilagus floridanus*) consumed less bark from paper birch seedlings grown under elevated CO_2_. They attributed this response to a measured increase in defensive compounds (i.e., terpenoids and condensed tannins); however, other indices of plant quality (e.g., C:N, lignin, etc.) were not reported. Research conducted a year earlier by the same research group [60] and under similar conditions did not show a feeding response by cottontail rabbits or field voles (*Microtus agrestis*), or a CO_2_ response in defensive compound concentration. Many studies have assessed the ability of gut microbes (harvested from the rumen of agricultural animals) to digest forage grown under either elevated CO_2_ or O_3_. These studies often show reduced IVDMD in response to the fumigation treatments, suggesting the possibility of reduced performance of ruminant animals in future atmospheres [14],[23],[24].

Several studies suggest that voles are a useful indicator species for the effects of forage quality on ruminant production [33],[34],[65]. It would be difficult, if not economically prohibitive, to implement a growth study to determine the effects of GAC using large mammalian herbivores (e.g., cervids, bovids), primarily because the infrastructure needed to produce the necessary plant biomass for such a study would be a monumental undertaking. Voles, however, eat much less, and their digestive capability [33] and productivity [34] are similar to that of much larger herbivores. The suggestion that voles are a good surrogate for feeding studies with ruminants has been advanced previously [34] and we reiterate that perspective here in the context of understanding the impacts of GAC on mammalian herbivores. Even when using voles, however, the resources needed for well replicated GAC studies remain non-trivial; in this study, the plant biomass available from each FACE ring limited our sample size to 1–2 replicate voles of each sex per replicate FACE ring, and the trial length to one week.

Despite their small size, voles are capable of impacting the composition and abundance of plants in grassland systems [66], influencing ecosystem-level processes such as nutrient cycling [67], and contributing to the input or removal of resources at levels comparable to much larger herbivores [67]–[69]. Furthermore, voles are a common dietary component for an array of predators [70]. As such, voles often play a central role in the structure and functioning of grassland ecosystems, particularly when they occur at intermediate to high densities. Reduced growth rate of voles, particularly at the weanling stage, could impact predation risk as well as the timing of several behavioral and developmental benchmarks, including dispersal, reproductive maturity, and late-season energy storage. If our observations reflect the potential effects of GAC on individuals in natural populations, reductions in the growth rate of voles could reverberate through ecosystems at several levels of organization.

Although we provided voles a choice of two species during the feeding trial, their diet in natural settings is more diverse [29],[31]. Further, their responses to the plants we harvested from Aspen FACE represent a snapshot in time in relation to plant chemistry. Given the considerable variability in the chemical response of plants to atmospheric change, both among species and through time, future work should focus on providing a more realistic number of plants harvested throughout the growing season to understand more fully the ability of mammals to compensate for GAC-mediated changes in plant quality.

## Conclusions

In conclusion, we show that global atmospheric change has the potential to affect the performance of a mammalian herbivore through changes in plant chemistry. The effect of CO_2_ was limited to the fiber fractions of plants, whereas ozone had strong and negative effects on plant quality. Experimental diets from elevated O_3_ rings reduced the growth rate of female voles. The growth rate of males, however, was unaffected by GAC-mediated changes in plant chemistry_._ The effects of global atmospheric change on phytochemistry will alter the growth of mammalian herbivores if they are unable to compensate for a general decrease in plant quality. Changes in herbivore performance could manifest as changes in plant community composition and ultimately ecosystem structure and function.

## Supporting Information

Figure S1 (DOCX)

## References

[1] Solomon S, Qin D, Manning M, Chen Z, Marquis M, et al., editors. (2007) Climate Change 2007: The Physical Science Basis. Contribution of Working Group I to the Fourth Assessment Report of the Intergovernmental Panel on Climate Change. New York: Cambridge University Press. 996 p.

[2] Stiling P, Cornelissen T (2007) How does elevated carbon dioxide (CO_2_) affect plant–herbivore interactions? A field experiment and meta-analysis of CO_2_-mediated changes on plant chemistry and herbivore performance. Glob Change Bio 13: 1823–1842.

[3] Valkama E, Koricheva J, Oksanen E (2007) Effects of elevated O_3_, alone and in combination with elevated CO_2_, on tree leaf chemistry and insect herbivore performance: a meta-analysis. Glob Change Bio 13: 184–201.

[4] Lindroth RL (2010) Impacts of elevated atmospheric CO_2_ and O_3_ on forests: phytochemistry, trophic interactions, and ecosystem dynamics. J Chem Ecol 36: 2–21. 20054619 10.1007/s10886-009-9731-4

[5] Bidart-Bouzat MG, Imeh-Nathaniel A (2008) Global change effects on plant chemical defenses against insect herbivores. J Integr Plant Bio 50: 1339–1354. 19017122 10.1111/j.1744-7909.2008.00751.x

[6] Wittig VE, Ainsworth EA, Naidu SL, Karnosky DF, Long SP (2009) Quantifying the impact of current and future tropospheric ozone on tree biomass, growth, physiology and biochemistry: a quantitative meta-analysis. Glob Change Bio 15: 396–424.

[7] Lindroth RL (2012) Atmospheric change, plant secondary metabolites, and ecological interactions. In: Iason GR, Dicke M, Hartley SE, editors. The Ecology of Plant Secondary Metabolites: From Genes to Global Processes. Ecological Reviews. Cambridge: Cambridge University Press. pp. 120–153.

[8] Bolsinger M, Lier ME, Hughes PR (1992) Influence of ozone air pollution on plant-herbivore interactions. Part 2: Effects of ozone on feeding preferences, growth, and consumption rates of monarch butterflies (*Danaus plexippus*). Environ Pollut 77: 31–37. 15091975 10.1016/0269-7491(92)90155-4

[9] Bender J, Muntifering RB, Lin JC, Weigel HJ (2006) Growth and nutritive quality of *Poa pratensis* as influenced by ozone and competition. Environ Pollut 142: 109–115. 16290915 10.1016/j.envpol.2005.09.012

[10] Gonzalez-Fernandez I, Bass D, Muntifering R, Mills G, Barnes J (2008) Impacts of ozone pollution on productivity and forage quality of grass/clover swards. Atmos Environ 42: 8755–8769.

[11] Howell RK, Smith LW (1977) Effects of ozone on nutritive quality of alfalfa. J Dairy Sci 60: 924–928.

[12] Powell MC, Muntifering RB, Lin JC, Chappelka AH (2003) Yield and nutritive quality of sericea lespedeza (*Lespedeza cuneata*) and little bluestem (*Schizachyrium scoparium*) exposed to ground-level ozone. Environ Pollut 122: 313–322. 12547521 10.1016/s0269-7491(02)00331-7

[13] Lewis JS, Ditchkoff SS, Lin JC, Muntifering RB, Chappelka AH (2006) Nutritive quality of big bluestem (*Andropogon gerardii*) and eastern gamagrass exposed to tropospheric ozone. Rangel Ecol Manag 59: 267–274.

[14] Muntifering RB, Chappelka AH, Lin JC, Karnosky DF, Somers GL (2006) Chemical composition and digestibility of *Trifolium* exposed to elevated ozone and carbon dioxide in a free-air (FACE) fumigation system. Funct Ecol 20: 269–275.

[15] Szantoi Z, Chappelka AH, Muntifering RB, Somers GL (2007) Use of ethyhlenediurea (EDU) to ameliorate ozone effects on purple coneflower (*Echinacea purpurea*). Environ Pollut 150: 200–208. 17412467 10.1016/j.envpol.2007.01.020

[16] Szantoi Z, Chappelka AH, Muntifering RB, Somers GL (2009) Cutleaf coneflower (*Rudbeckia laciniata* L.) response to ozone and ethylenediurea (EDU). Environ Pollut 157: 840–846. 19084304 10.1016/j.envpol.2008.11.014

[17] Frei M, Makkar HPS, Becker K, Wissuwa M (2010) Ozone exposure during growth affects the feeding value of rice shoots. Anim Feed Sci Tech 155: 74–79.

[18] Batzli GO, Broussard AD, Oliver RJ (1994) The integrated processing response in herbivorous small mammals. In: Chivers DJ, Langer P, editors. The digestive system in mammals: food, form, and function. Cambridge: Cambridge University Press. pp 324–35.

[19] Young Owl M, Batzli GO (1998) The integrated processing response of voles to fibre content of natural diets. Funct Ecol 12: 4–13.

[20] Sakaguchi E (2003) Digestive strategies of small hindgut fermenters. Anim Sci J 74: 327–337.

[21] Zvereva EL, Kozlov MV (2006) Consequences of simultaneous elevation of carbon dioxide and temperature for plant-herbivore interactions: a meta-analysis. Glob Change Bio 12: 27–41.

[22] Akin DE, Kimball BA, Windham WR, Pinter PJ, Wall GW, et al (1995) Effect of free-air CO_2_ enrichment (FACE) on forage quality of wheat. Anim Feed Sci and Tech 53: 29–43.

[23] Carter EB, Theodorou MK, Morris P (1999) Response of *Lotus corniculatus* to environmental change. 2. Effect of elevated CO_2_, temperature and drought on tissue digestion in relation to condensed tannin and carbohydrate accumulation. J Sci Food Agric 79: 1431–1440.

[24] Morgan JA, Mosier AR, Milchunas DG, LeCain DR, Nelson JA, et al (2004) CO_2_ enhances productivity, alters species composition, and reduces digestibility of shortgrass steppe vegetation. Ecol App 14: 208–219.

[25] Milchunas DG, Mosier AR, Morgan JA, LeCain DR, King JY, et al (2005) Elevated CO_2_ and defoliation effects on a shortgrass steppe: Forage quality versus quantity for ruminants. Agric Ecosyst Environ 111: 166–184.

[26] Derting TL, Bogue BA (1993) Responses of the gut to moderate energy demands in a small herbivore (*Microtus pennsylvanicus*). J Mammal 74: 59–68.

[27] Wiggins NL, McArthur C, Davies NW (2006) Diet switching in a generalist mammalian folivore: fundamental to maximizing intake. Oecologia 147: 650–657. 16328546 10.1007/s00442-005-0305-z

[28] Naya DE, Karasov WH, Bozinovic F (2007) Phenotypic plasticity in laboratory mice and rats: a meta-analysis of current ideas on gut size flexibility. Evol Ecol Res 9: 1363–1374.

[29] Batzli GO (1985) Nutrition. In: Tamarin RH, editor. Biology of New World *Microtus*. Shippensburg, PA: American Society of Mammalogists. pp. 779–811.

[30] Bergeron J-M, Jodoin L (1993) Intense grazing by voles (*Microtus pennsylvanicus*) and its effects on habitat quality. Can J Zool 71: 1823–1830.

[31] Marquis RJ, Batzli GO (1989) Influence of chemical factors on palatability of forage to voles. J Mammal 70: 503–511.

[32] Carleton MD (1985) Macroanatomy. In: Tamarin RH, editor. Biology of New World *Microtus*. Shippensburg, PA: American Society of Mammalogists. pp. 779–811.

[33] Keys JE, Van Soest PJ (1970) Digestibility of forages by the meadow vole (*Microtus pennsylvanicus*). J Dairy Sci 53: 1502–1508.

[34] Shenk JS, Barnes RF, Donker JD, Marten GC (1975) Weanling meadow vole and dairy cow response to alfalfa hay. Agron J 67: 569–571.

[35] Castle KT, Wunder BA (1995) Limits to food intake and fiber utilization in the prairie vole, *Microtus ochrogaster*: effects of food quality and energy need. J Comp Physiol B 164: 609–617.

[36] Lindroth RL, Osier TL, Wood SA, Barnhill HRA (2001) Effects of genotype and nutrient availability on phytochemistry of trembling aspen (*Populus tremuloides* Michx.) during leaf senescence. Biochem Syst Ecol 30: 297–307.

[37] DeGabriel JL, Wallis IR, Moore BD, Foley WJ (2008) A simple, integrative assay to quantify nutritional quality of browses for herbivores. Oecologia 156: 107–116. 18288495 10.1007/s00442-008-0960-y

[38] Beacham TD (1979) Survival in fluctuating populations of the vole *Mictotus townsendii* . Can J Zool 57: 2375–2384.

[39] Desy EA, Thompson EF (1983) Effects of supplemental food of a *Mictotus pennsylvanicus* population in Central Illinois. J Anim Ecol 52: 127–140.

[40] Gaines MS, Johnson ML (1984) A multivariate study of the relationship between dispersal and demography in populations of *Mictotus ochrogaster* in Eastern Kansas. Am Midl Nat 111: 223–233.

[41] Koteja P, Weiner J (1993) Mice, voles, and hamsters: metabolic rates and adaptive strategies in muroid rodents. Oikos 66: 505–514.

[42] Filion M, Dutilleul P, Potvin C (2000) Optimum experimental design for Free-Air Carbon dioxide Enrichment (FACE) studies. Glob Chang Bio 6: 843–854.

[43] Quinn GP, Keough MJ (2002) Experimental design and data analysis for biologists. Cambridge: Cambridge University Press. 537 p.

[44] Carrascal LM, Galvan I, Gordo O (2009) Partial least squares regression as an alternative to current regression methods used in ecology. Oikos 118: 681–690.

[45] Adnan N, Ahmad MH, Adnan R (2006) A comparative study on some methods of handling multicollinearity problems. Matematika 22: 109–119.

[46] Wold S, Ruhu H, Wold H (1984) The collinearity problem in linear regression: the partial least squares approach to generalized inverses. SIAM J Sci Stat Comp 5: 735–743.

[47] Wold S (1995) PLS for multivariate linear modeling. In: Van de Waterbeemd H (ed) Chemometric methods in molecular design. VCH Verlagsgesellschaft mbH, Weinheim, Germany. pp 195–218.

[48] Fritschi FB, Boote KJ, Sollenberger LE (1999) Carbon dioxide and temperature effects of forage establishment: tissue composition and nutritive value. Glob Chang Bio 5: 743–753.

[49] Lilley JM, Bolger TP, Peoples MB, Gifford RM (2001) Nutritive value and the nitrogen dynamics of *Trifolium subterraneum* and *Phalaris aquatica* under warmer, high-CO_2_ conditions. New Phytol 150: 385–395.

[50] Owensby CE, Cochran RM, Auen LM (1996) Effects of elevated carbon dioxide on forage quality for ruminants. In: Korner C, Bazzaz FA, editors. Carbon dioxide, populations, and communities. San Diego, CA: Academic Press. Pp. 363–371.

[51] Schadler M, Roeder M, Brandl R, Matthias D (2007) Interacting effects of elevated CO_2_, nutrient availability and plant species on a generalist invertebrate herbivore. Glob Change Bio 13: 1005–1015.

[52] Bazzaz FA, Williams WE (1991) Atmospheric CO_2_ concentrations within a mixed forest: implications for seedling growth. Ecology 72: 12–16.

[53] Kangasjärvi J, Talvinen J, Utriainen M, Karjalainen R (1994) Plant defense systems induced by ozone. Plant Cell Environ 17: 783–794.

[54] Hammond KA, Wunder BA (1991) The role of diet quality and energy need in the nutritional ecology of a small herbivore, *Microtus ochrogaster* . Physiol Zoo 54: 541–567.

[55] Gross JE, Wang Z, Wunder BA (1985) Effects of food quality and energy needs: changes in gut morphology and capacity of *Micrtotus ochrogaster* . J Mammal 66: 661–667.

[56] Trier TM (1996) Diet-induced thermogenesis in the prairie vole, *Microtus ochrogaster* . Physiol Zoo 69: 1456–1468.

[57] Ditchkoff SS, Boyd CS, Welch ER, Raglin JB, Lochmiller RL (1998) Nitrogen requirements of the adult prairie vole (*Mictotus ochrogaster*). Am Mid Nat 140: 387–392.

[58] Voltura MB, Wunder BA (1998) Effects of ambient temperature, diet quality, and food restriction on body composition dynamics of the prairie vole, *Microtus ochrogaster* . Physiol Zoo 71: 321–328. 10.1086/515929 9634179

[59] Batzli GO (1986) Nutritional ecology of the California vole: effects of food quality on reproduction. Ecology 67: 406–412.

[60] Karasov WH, Martinez del Rio C (2007) Physiological Ecology. Princeton: Princeton University Press. 741 p.

[61] Lindroth RL, Batzli GO, Guntenspergen GR (1984) Artificial diets for use in nutritional studies with microtine rodents. J Mammal 65: 139–143.

[62] Voltura MB (1997) Seasonal variation in body composition and gut capacity of the prairie vole (*Microtus ochrogaster*). Can J Zoo 75: 1714–1719.

[63] Kuokkanen KP, Niemelä J, Matala R, Julkunen-Tiitto R, Heinonen J, et al (2004) The effects of elevated CO_2_ and temperature on the resistance of winter-dormant birch seedlings (*Betula pendula*) to hares and voles. Glob Change Bio 10: 1504–1512.

[64] Mattson WJ, Kuokkanen K, Niemelä P, Julkunen-Tiitto R, Kellomäki S, et al (2004) Elevated CO_2_ alters birch resistance to Lagamorpha herbivores. Glob Change Bio 10: 1402–1413.

[65] Russo SL, Shenk JS, Barnes RF, Moore JE (1981) The weanling meadow vole as a bioassay of forage quality of temperate and tropical grasses. J Anim Sci 52: 1205–1210.

[66] Howe HF, Zorn-Arnold B, Sullivan A, Brown JS (2006) Massive and distinctive effects of meadow voles on grassland vegetation. Ecology 87: 3007–3013. 17249225 10.1890/0012-9658(2006)87[3007:madeom]2.0.co;2

[67] Sirotnak JM, Huntly NJ (2000) Direct and indirect effects of herbivores on nitrogen dynamics: voles in riparian areas. Ecology 81: 78–87.

[68] Clark JE, Hellgren EC, Parsons JL, Jorgenson EE, Engle DM, et al (2005) Nitrogen outputs from fecal and urine deposition of small mammals: implication for nitrogen cycling. Oecologia 144: 447–455. 15942760 10.1007/s00442-005-0004-9

[69] Habeck CW, Meehan TD (2008) Mass invariance of population nitrogen flux by terrestrial mammalian herbivores: an extension of the energetic equivalence rule. Ecol Lett 11: 898–903. 18445029 10.1111/j.1461-0248.2008.01198.x

[70] Pearson OP (1985) Predation. In: Tamarin RH, editor. Biology of New World *Microtus*. Shippensburg, PA: American Society of Mammalogists. pp. 779–811.

