# Supplementary material for: Influence of Global Atmospheric Change on the Feeding Behavior and Growth Performance of a Mammalian Herbivore, Microtus ochrogaster
Source: PLoS One. 2013 Aug 19;8(8):e72717. doi: 10.1371/journal.pone.0072717 (PMC3747069; doi:10.1371/journal.pone.0072717)
Supplement: Figure S1 — (DOCX) [file pone.0072717.s001.docx]

**Supporting Information:** Validation of partial least squares regression (PLSR) models.

**Figure S1.** Observed versus predicted values from PLSR final models for a) male and b) female consumption rate and c) male and d) female growth rate in relation to variation in plant phytochemistry.

**

**
